# Effect of vitamin K on wound healing: A systematic review and meta-analysis based on preclinical studies

**DOI:** 10.3389/fphar.2022.1063349

**Published:** 2022-12-02

**Authors:** Saiqing Tang, Zhen Ruan, Axue Ma, Dong Wang, Jiushe Kou

**Affiliations:** ^1^ Second School of Clinical Medicine, Shaanxi University of Chinese Medicine, Xianyang, China; ^2^ Xianyang Central Hospital, Xianyang, China; ^3^ Medical Research and Experiment Center, Shaanxi University of Chinese Medicine, Xianyang, China; ^4^ Shaanxi Key Laboratory of Research on TCM Physical Constitution and Disease Prevention and Treatment, Xianyang, China; ^5^ Second Affiliated Hospital of Shaanxi University of Chinese Medicine, Xianyang, China

**Keywords:** vitamin K, wound healing, wound, meta-analysis, systematic review

## Abstract

**Background:** In recent years, many studies have found that vitamin K is beneficial to wound healing. However, some research results seem to be in conflict. The purpose of this study was to evaluate the effect of vitamin K on wound healing.

**Methods:** We systematically and comprehensively searched the PubMed, Web of Science, Embase, Cochrane library, China National Knowledge Infrastructure (CNKI), VIP and Wanfang eletronic databases. We applied revman5.3 software to calculate the weighted mean difference (WMD) of 95% confidence interval (CI) of animal and cell groups to evaluate the effect of vitamin K on wound healing. Two researchers independently selected studies and used the Cochrane Collaboration tool to assess the risk of bias in the included studies. The overall quality of evidence was assessed using the Recommendation, Assessment, Development and Evaluation (GRADE) working group approch.

**Results:** Among the 1081 articles searched, 6 articles (16 studies in total) met the inclusion criteria. The results of quantitative analysis showed that vitamin K was beneficial to increase the wound healing rate in animal models [rat model: WMD = 27.45 (95% CI: 13.46, 41.44); *p* = 0.0001], but the opposite result was obtained in cell experiments [WMD = −33.84 (95% CI: −56.90, −10.79); *p* = 0.004].

**Conclusion:** This meta-analysis hits that vitamin K could affect the process of wound healing, especially in animal models. While we could not know the clear role at present, which requires larger scale research. In addition, the concentration and safe dose of vitamin K also deserve further study.

## Introduction

Wounds are defined as the disruption or interruption of the normal anatomical structure and functional continuity of tissue (Farahani and Shafiee, 2021). Wound healing is an important physiological process of self-repair and regeneration after injury, which includes three consecutive and overlapping stages: hemostasis/inflammation, proliferation, and remodeling (Kim and Nair, 2019). The destruction of normal wound healing will lead to the formation of refractory ulcer, whereas excessive healing will lead to the formation of keloid, which seriously damages the appearance and normal function of the human body and increases the economic and medical burden of the society and country (Wang et al., 2018; Subramaniam et al., 2021). Therefore, effective wound management and treatment are important. Vitamin K, a fat-soluble vitamin, is an essential cofactor of γ-glutamyl carboxylase, which is involved in the catalytic carboxylation of specific glutamic acid residues. Vitamin K primarily includes vitamin K1 (phylloquinone), vitamin K2 (menaquinones), vitamin K3 (menadione), and other forms, among which the former two are natural forms, whereas the latter is synthetic (Halder et al., 2019). Vitamin K is commonly used in the treatment of various diseases, including vascular calcification, osteoporosis, diabetes, and liver cancer, because of its pro-coagulant, anti-inflammatory, antioxidant, and other pharmacological effects (Anwar et al., 2019; Fusaro et al., 2020; Shioi et al., 2020; Popa et al., 2021). In addition, vitamin K plays an important role in wound healing (Pazyar et al., 2019). Considering the lack of comprehensive quantitative analyses of the effect of vitamin K on wound healing, we reviewed relevant *in vivo* and *in vitro* studies to evaluate the effect of vitamin K on wound healing and provide insights into clinical treatment and new drug development.

## Materials and methods

We used PRISMA 2020 (Preferred Reporting Items for Systematic Reviews and Meta-Analyses) and AMSTAR 2 as guidelines for selecting relevant studies and assessing the methodological quality of systematic reviews (Page et al., 2021).

### Search strategy

Two independent researchers conducted a thorough literature search. We used English (PubMed, Web of Science, Embase, the Cochrane Library) and Chinese [China National Knowledge Infrastructure (CNKI), VIP, Wanfang] electronic databases to search relevant studies published before March 2022. The following subject terms were used for the search: “wound healing” OR “injury repair” OR “regeneration” OR “wound epithelialization” OR “injury repair” AND “Vitamin K” OR “Vitamin K1” OR “Vitamin K2” OR “Vitamin K3” OR “Phylloquinone” OR “Menaquinone” OR “menadione”. The language of the study was restricted to English and Chinese. A detailed search strategy is shown in Supplementary Material S1.

### Study selection

The inclusion criteria were as follows: 1) the selected studies must be randomized controlled trials with relevant original data; 2) treatment with vitamin K during wound healing to evaluate the effect of vitamin K on wound healing; 3) comparison was made between the no-treatment group or other drug treatment such as ethanol or phenytoin sodium; 4) the study should have appropriate efficacy indicators, including wound contraction rate, tensile strength, and healing time.

The exclusion criteria were as follows: 1) meetings, meta-analysis articles, or reviews should be excluded; 2) it is not related to the effect of vitamin K on wound healing; 3) duplicate data, incomplete data or no original data provided; 4) data of wound contraction rate, tensile strength, or healing days were not provided;

### Data extraction and quality assessment

Two researchers (Saiqing Tang and Dong Wang) independently reviewed the title and abstract of the study in accordance with the inclusion criteria and exclusion criteria. For potentially relevant studies, the full text was obtained and further reviewed. The data of the included articles were extracted into a standardized table, including the first author’s name, year, country, species, age/weight, the number of samples in the control and experimental groups, and intervention measures of each group. The differences arising in the process were discussed and resolved with the third researcher (Zhen Ruan). For animal model studies, wound contraction percentage (%) and wound tensile strength were extracted on the 7th day after injury. For cell experiment studies, the percentage of wound contraction (%) was extracted at the 18th hour after modeling, and the most recent sampling time was used if the 7th day or 18th h data were not published. If the data in the article were not in the form of wound healing rates, then simple arithmetic must be applied. The same two researchers used the Cochrane bias risk assessment tool to independently assess the following bias risk factors of all included studies: random sequence generation, allocation concealment, blinding of subjects and researchers, blinding of outcome evaluators, incomplete outcome data, and selective reporting and other bias. The determination of each item was divided into three levels: low risk, unclear risk, or high risk. Considering the differences between animal studies and randomized controlled trials, we adopted the SYRCLE’s risk of bias tool based on the Cochrane Collaboration risk of bias tool to evaluate the quality of animal studies (Hooijmans et al., 2014). Any differences were discussed and resolved with the third researcher (Zhen Ruan).

### Data analysis

All study data were analyzed using ReviewManager5.3 and generated corresponding forest plots. Continuous data were assessed by weighted mean difference (WMD) with 95% confidence intervals (95%CI). The heterogeneity of the included studies was assessed using Q and I^2^ statistics, with I^2^ scores of 25%, 50%, and 75% corresponding to low, medium, and high heterogeneity, respectively. Effect sizes were pooled using random effect models or fixed effect models. If I^2^ < 50% and *p* ≥ 0.1, then the fixed effect model was used to calculate the combined effect size. Otherwise, a random effect model was used.

### Certainty in the evidence

We used the Grades of Recommendation, Assessment, Development and Evaluation (GRADE) working group approch to grade the quality of evidence. The quality of evidence is divided into four levels: high, moderate, low and very low. In GRADE approch, randomized controlled trials (RCTs) without serious defects are high-quality evidence. If there is a serious or very serious risk of bias, inconsistency, indirectness, imprecision and publication bias, the quality of evidence will be degraded accordingly (Puhan et al., 2014).

## Results

### Study selection and characteristics

**FIGURE 1:**
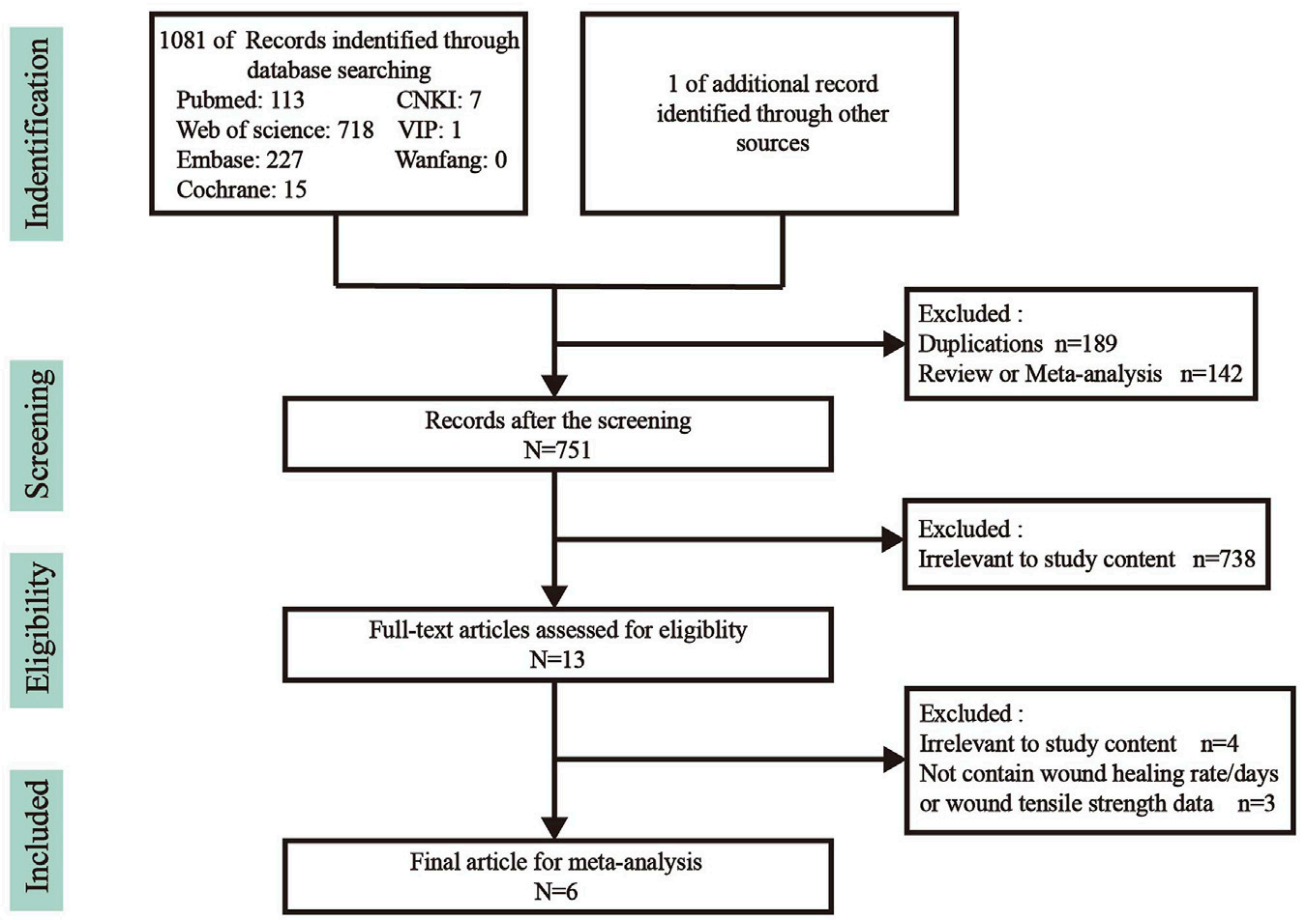
Flowchart of study search.

The flow chart of study selection is shown in Figure 1. In brief, we obtained 1082 articles through search, including 113 from PubMed, 718 from Web of Science, 227 from Embase, 15 from Cochrane, seven from CNKI, one from VIP and one of additional record from other sources. A total of 189 and 142 articles were excluded because of duplication and review/meta-analysis, respectively. After screening the titles and abstracts, 738 articles unrelated to the effect of vitamin K on wound healing were excluded, leaving 13 articles for further screening. By reading the full text for screening, seven articles were excluded (Supplementary Material S5) for the following reasons: four articles were not related to the effect of vitamin K on wound healing, and three articles did not report the wound healing rate, healing days, or tensile strength. Finally, six articles (including 16 studies) were eligible for meta-analysis (Amaral et al., 2014; Hemmati et al., 2014; Pinilla et al., 2014; Rush et al., 2016; Pazyar et al., 2019; Kandhasamy et al., 2021). Detailed characteristics of the included studies are shown in Table 1. The studies were published from 2014 to 2021 in five countries: Brazil, Iran, Spain, the United States, and China. Among the 16 studies, seven and one studies used rat and mouse models, respectively, and six and two studies were obtained from cellular and clinical trials, respectively. Male albino rats, SD rats, and Wistar rats were used in rat model studies. The cells used in six *in vitro* studies were all obtained from human. In two clinical studies conducted by Pazyar et al. (2019), a total of 54 patients were enrolled, and all participants were randomly assigned to either the control or intervention groups. Hemmati et al. (2014), Rush et al. (2016), Pinilla et al. (2014) applied different concentrations of vitamin K for intervention. In this meta-analysis, studies with vitamin K as the intervention method were included, ignoring the form and adjuvant of vitamin K. For example, in the study of Kandhasamy et al. (2021), silk fibroin electrospun fibers in vitamin K were considered in the intervention method. The included studies used Eucerin, 1% phenytoin, phosphate-buffered saline (PBS), etc. As controls, whereas blank controls were used in the studies of Amaral et al. (2014)) and Pinilla et al. (2014)

**TABLE 1:** Characteristics of studies used in meta-analysis.

Studies	Species	Age/Weight	Sample size	Intervention	Control	Outcome measurement	Sampling time	Country
Hemmati et al.2014 (1)	albino rats	150–170 G	Ne = 8	1%VK1+ eucerin	eucerin	WSR(mean ± SE)	7 days	Iran
	(M)		Nc = 8			/WTS		
Hemmati et al.2014 (2)	albino rats	150–170 G	Ne = 8	2%VK1+ eucerin	eucerin	WSR(mean ± SE)	7 days	Iran
	(M)		Nc = 8			/WTS		
Hemmati et al.2014 (3)	albino rats	150–170 G	Ne = 8	1%VK1+ eucerin	1% phenytoin	WSR(mean ± SE)	7 days	Iran
	(M)		Nc = 8		+eucerin	/WTS		
Hemmati et al.2014 (4)	albino rats	150–170 G	Ne = 8	2%VK1+ eucerin	1% phenytoin	WSR(mean ± SE)	7 days	Iran
	(M)		Nc = 8		+eucerin	/WTS		
Kandhasamy et al. (2021) (1)	SD rats	6–7 W/	Ne = 6	SF-VKC	SF	WHR	6 days	China
	(M)	300−350G	Nc = 6			(mean ± SD)		
Kandhasamy et al. (2021) (2)	SD rats	6–7 W/	Ne = 6	SF-VKC	PBS	WHR	6 days	China
	(M)	300−350G	Nc = 6			(mean ± SD)		
Amaral et al. (2014)	Wistar rats	320–410G	Ne = 4	Vitamin K1	no treat	WTS	7 days	Brazil
	(M)		Nc = 4			(mean ± SE)		
Rush et al. (2016) (1)	C57Bl6 mice	8–10 W	—	0.3 μM VK3+	10 μM RTKi	WHR	16 h	United States
	(F)		—	10 μM RTKi		(mean ± SE)		
Rush et al. (2016) (2)	hTCE-pi cells	—	Ne = 4	0.3 μM VK3+	0.3 μM RTKi	WHR	4.5 h	United States
		—	Nc = 4	0.3 μM RTKi		(mean ± SE)		
Rush et al. (2016) (3)	hTCE-pi cells	—	Ne = 4	3 μM VK3+	0.3 μM RTKi	WHR	4.5 h	United States
		—	Nc = 4	0.3 μM RTKi		(mean ± SE)		
Pinilla et al. (2014) (1)	HCF	—	Ne = 12	1 mg/L VK3	no treat	WAS	18 h	Spain
		—	Nc = 12			(mean ± SD)		
Pinilla et al. (2014) (2)	HCF	—	Ne = 12	2 mg/L VK3	no treat	WAS	18 h	Spain
		—	Nc = 12			(mean ± SD)		
Pinilla et al. (2014) (3)	HCF	—	Ne = 12	4 mg/L VK3	no treat	WAS	18 h	Spain
		—	Nc = 12			(mean ± SD)		
Pinilla et al. (2014) (4)	HCF	—	Ne = 12	6 mg/L VK3	no treat	WAS	18 h	Spain
		—	Nc = 12			(mean ± SD)		
Pazyar et al. (2019) (1)	Patients (B)	17.4–45.7Y	Ne = 16	1%VK1+ eucerin	eucerin	HD (mean ± SE)/WAS	--/7 days	Iran
	Patients		Nc = 19					
Pazyar et al. (2019) (2)	(B)	23.4–48.6Y	Ne = 16	1%VK1+ eucerin	1% phenytoin+ eucerin	HD (mean ± SE)	--/7 days	Iran
			Nc = 19			/WAS		

SD, Sprague-Dawley; hTCE-pi, human telomerase-immortalized corneal epithelial; HCF, human conjunctival fibroblasts; M,Male; F, Female; B, both sexes; G, Gram; W, weeks; Y, years; Ne, number of experiments; Nc, number of controls; VK, vitamin K; SF-VKC, vitamin K3 carnosine peptide incorporated silk fibroin; SF, silk fibroin; RTKi, receptor tyrosine kinase inhibitor; WSR, wound size rate; WTS, wound tensile strength; WHR, wound healing rate; WAS, wound areas surface; HD, healing days.

### Methodologies for the bias of selected studies

**FIGURE 2:**
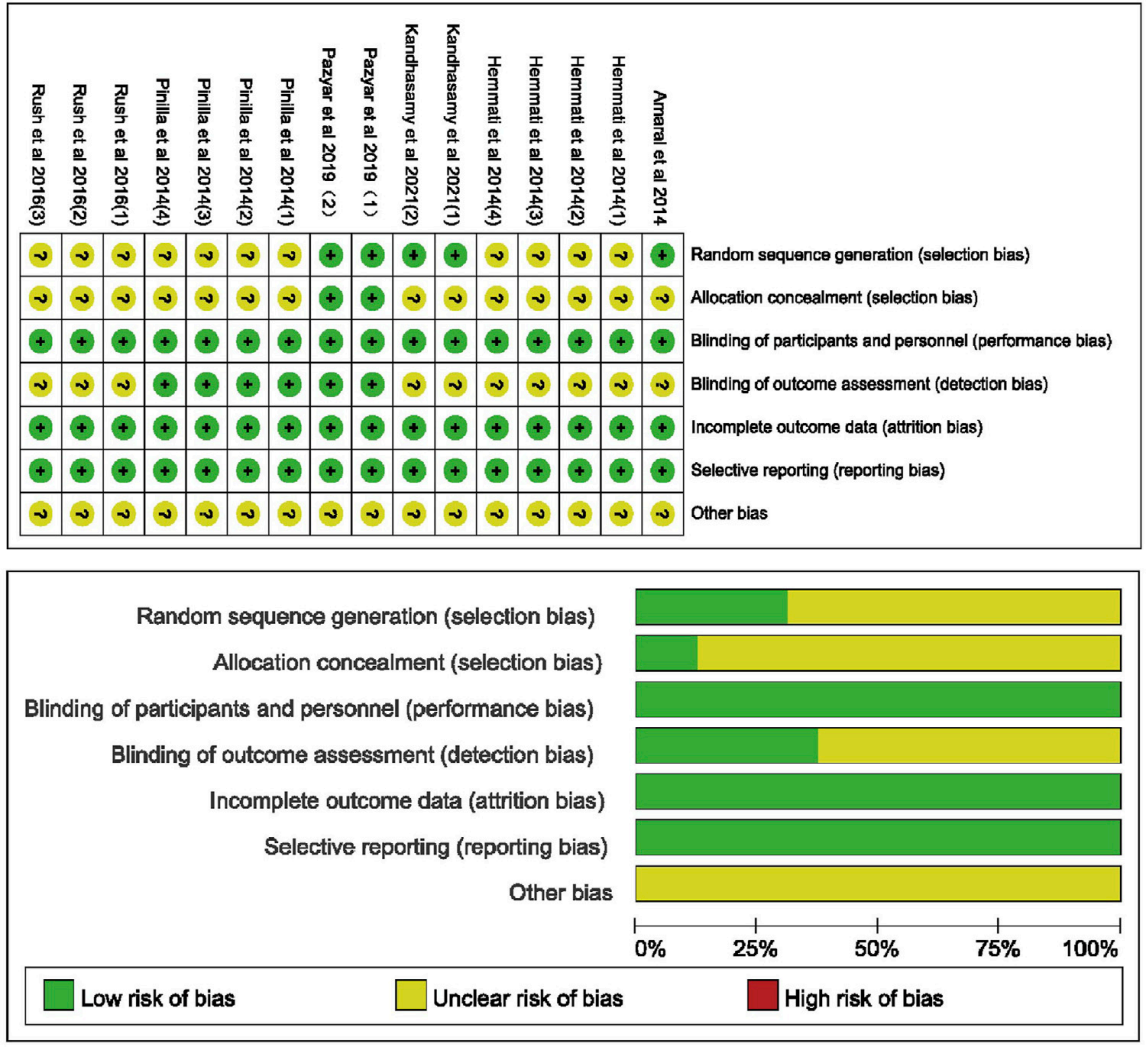
Risk of bias summary.

The risk of bias for included studies is summarized in Figure 2. The risk of bias for studies in animal models is summarized in Table 2. Of the 16 studies, five explicitly indicated the use of random sequence generation. All studies showed ambiguity about the allocation of hidden risks of bias, except for two studies conducted by Pazyar et al. (2019), which showed a low risk of bias. Two studies from clinical trials clearly showed a low risk of bias in blinding participants and experimentalists. Considering that animals and cells cannot understand the experiment, the risk of performance bias in animal and cell experiments is low. With regard to blinding outcome assessment, the six studies conducted by Pazyar et al. (2019) and Pinilla et al. (2014) showed a low risk of bias, and the remaining studies were not determined by us because of insufficient information. All studies showed low risks of bias for incomplete data and selective reporting, whereas other risks of bias were unclear.

**TABLE 2:** SYRCLE’s tool for assessing the risk of bias in animal studies.

Studies	*Was the allocation sequence adequately generated and applied?	Were the groups similar at baseline or were they adjusted for confounders in the analysis?	*Was the allocation adequately concealed?	Were the animals randomly housed during the experiment?	Were the caregivers and/or investigators blinded from knowledge which intervention each animal received during the experiment?	Were animals selected at random for outcome assessment?	Was the outcome assessor blinded?	*Were incomplete outcome data adequately addressed?	*Are reports of the study free of selective outcome reporting?	*Was the study apparently free of other problems that could result in high risk of bias?
Hemmati et al. (2014)	unclear	low	unclear	low	unclear	unclear	unclear	low	low	unclear
Hemmati et al. (2014)	unclear	low	unclear	low	unclear	unclear	unclear	low	low	unclear
Hemmati et al. (2014)	unclear	low	unclear	low	unclear	unclear	unclear	low	low	unclear
Hemmati et al. (2014)	unclear	low	unclear	low	unclear	unclear	unclear	low	low	unclear
Kandhasamy et al.(2021)	low	low	unclear	unclear	unclear	unclear	unclear	low	low	unclear
Kandhasamy et al.(2021)	low	low	unclear	unclear	Unclear	unclear	unclear	low	low	unclear
Amaral et al. (2014)	low	low	unclear	low	Unclear	unclear	unclear	Low	low	unclear
Rush et al. (2016)	unclear	low	unclear	unclear	Unclear	unclear	unclear	Low	low	unclear

*Items in agreement with the items in the Cochrane Risk of Bias tool.

### Results of the meta-analysis

#### Wound healing rate in animal model

**FIGURE 3:**
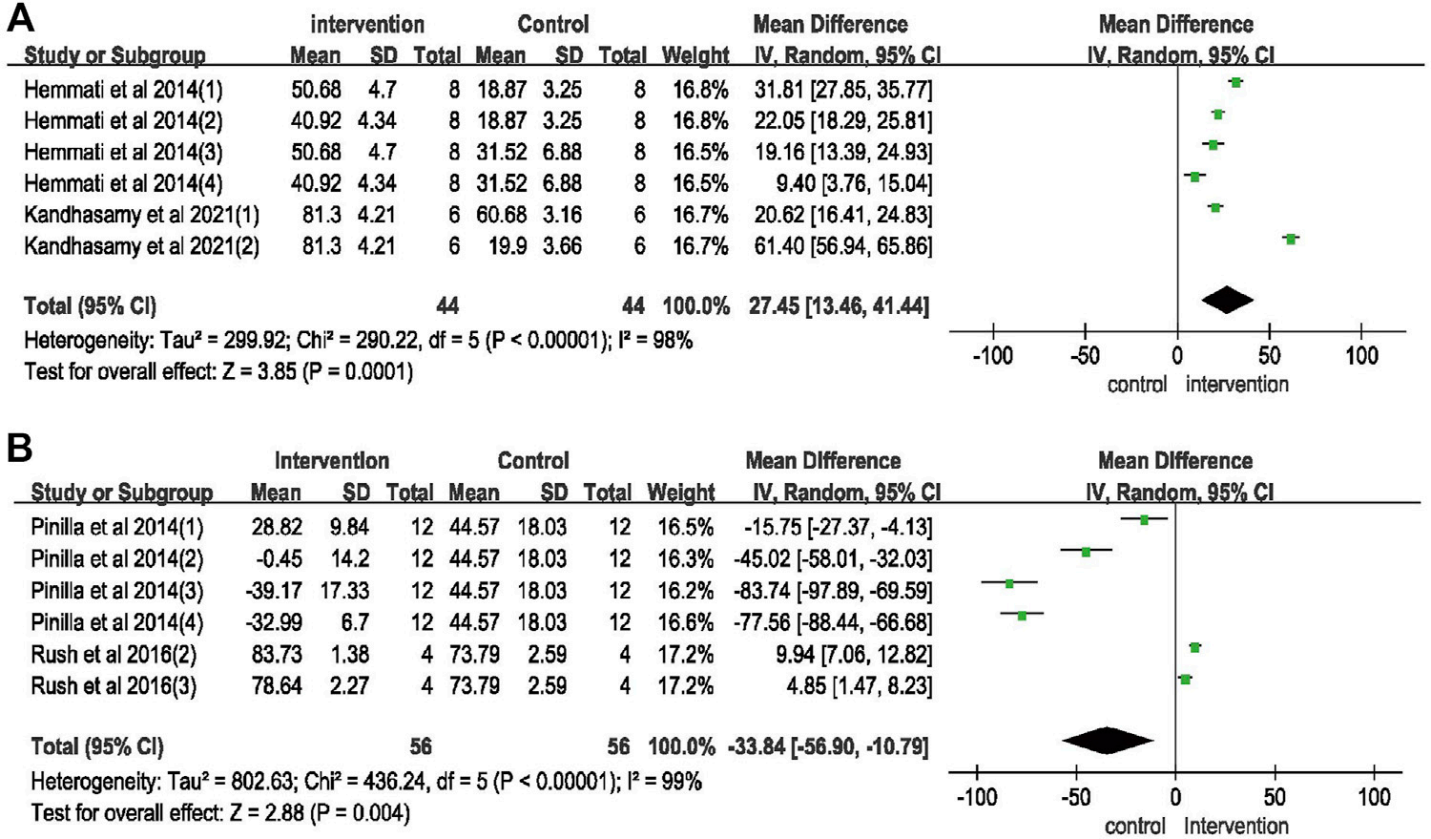
Meta-analysis on vitamin K for wound healing. **(A)** Wound healing rate on rat model; **(B)** wound healing rate on cell experiments.

Six rat model studies involved the effect of vitamin K on wound healing, including 22 rats in the vitamin K group and 28 rats in the control group. Pooled results (Figure 3A) suggested that vitamin K treatment was associated with an increased wound healing rate [WMD = 27.45 (95%CI: 13.46, 41.44)]. The heterogeneity of the combined results was also significant (I^2^ = 98%, *p* < 0.00001). The overall effect test (Z) was 3.85 (*p* = 0.0001).

#### Wound healing rate on cell experiments

We evaluated the effect of vitamin K on wound healing in six cell experiment studies, and the results (Figure 3B) suggested that the wound healing rate was decreased in the vitamin K intervention group [WMD = –33.84 [95%CI: −56.90, −10.79)]. The combined results showed significant heterogeneity (I^2^ = 99%, *p* < 0.00001). The overall effect test (Z) was 2.88 (*p* = 0.004).

#### Wound tensile strength in animal model

**FIGURE 4:**
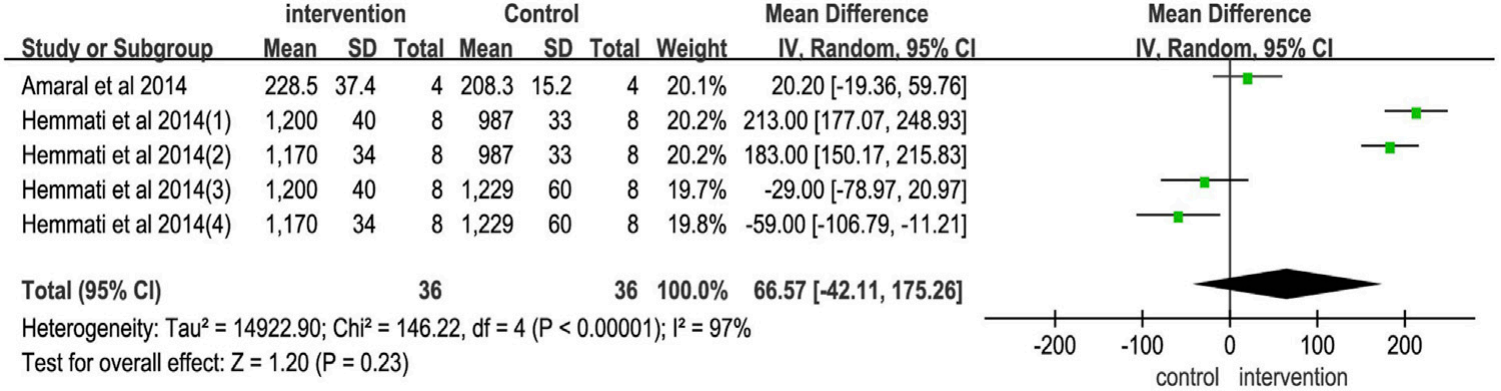
Meta-analysis on vitamin K for wound tensile strength.

Five rat model studies reported the wound tensile strength. The combined results (Figure 4) showed WMD (95% CI) of 66.57 (−42.11,175.26), I^2^ of 97% (*p* < 0.00001), and effect Z of 1.2 (*p* = 0.23). The results indicated no significant difference in wound tensile strength between the vitamin K group and control group.

### Grading the evidence

The evaluations of evidence quality according to GRADE approach are shown in Table 3. Due to the small sample size included in the studies and the serious inconsistency between different studies, the quality was degraded.

**TABLE 3:** Summary of findings table as per GRADE working group.

Outcomes	Parameters compared	No of participants (studies)	Quality of the evidence (GRADE)	Comments
wound healing rate (rats)	The mean wound healing rate (rats) in the intervention groups was **27.45 higher** ^c^ (13.46–41.44 higher)	88 (6 studies)	⊕⊕⊕⊝ **moderate** ^a^	MD 27.45 (13.46–41.44)
wound healing rate (cells)	The mean wound healing rate (cells) in the intervention groups was **33.84 lower** ^c^ (56.9–10.79 lower)	112 (6 studies)	⊕⊕⊝⊝ **low** ^a^ ^,^ ^b^	MD -33.84 (-56.9 to-10.79)
wound tensile strength	The mean wound tensile strength in the intervention groups was **66.57 higher** ^c^ (42.11 lower to 175.26 higher)	72 (5 studies)	⊕⊕⊝⊝ **low** ^a^ ^,^ ^b^	MD 66.57 (-42.11–175.26)
GRADE Working Group grades of evidence				
High quality: Further research is very unlikely to change our confidence in the estimate of effect				
Moderate quality: Further research is likely to have an important impact on our confidence in the estimate of effect and may change the estimate				
Low quality: Further research is very likely to have an important impact on our confidence in the estimate of effect and is likely to change the estimate				
Very low quality: We are very uncertain about the estimate				

^a^ The sample size included in the studies were small.

^b^ Certainty in the evidence downgraded by 1 level due to serious inconsistency.

^c^ They correlated with Figures 3, 4, higher means positve value and lower means negative value.

## Discussion

This study is a systematic compilation of the existing literature on the efficacy of vitamin K for wound healing. Through comprehensive and systematic search of literature and strict screening, we obtained six articles (16 qualified studies) in total. We divided these studies into the animal group, cell group, and clinical group. The animal group includes rat and mouse models. Only one article is found in mouse model and clinical test; thus, comprehensive quantitative analysis cannot be carried out. This study only summarizes the research results of the rat model and cell test. The intervention method used vitamin K to cure wound, and it was compared with PBS, conventional medicine, or blank group. The meta-analysis results of the animal model group suggested that vitamin K may improve the wound healing rate. However, the cell group showed opposite summary analysis results, which may be due to the small number of included studies, the quality of the literature, and the high heterogeneity. The heterogeneity of rat models is significant, with I^2^ of 98% (*p* < 0.00001), probably because only six studies were included in this group. Four studies conducted by Hemmati et al. (2014) explored the effect of Eucerin ointment containing 1% vitamin K1 or 2% vitamin K1 on wound contraction rate relative to Eucerin ointment or Eucerin ointment containing 1% phenytoin. The difference in vitamin K concentration and control group may also be the source of high heterogeneity. In addition, the different forms and adjuvants of vitamin K will cause high heterogeneity. In the two studies conducted by Kandhasamy et al. (2021), vitamin K was brought into silk fibroin electrospun fibers and compared with silk fibroin electrospun fibers and PBS. In these studies, the strain and body weight of rats may also cause heterogeneity. However, regardless of the study excluded, the overall results have not changed in direction, which shows that the summarized results are reliable. In view of the high heterogeneity of the rat model, we conducted a subgroup analysis. Among the four studies conducted by Hemmati et al. (2014), 1% vitamin K studies has the most significant effect. In order to compare the effect of vitamin K on wound healing, an common drug is better to use as an control group, the study of Hemmati et al. (2014) 3) with Euserine ointment containing 1% phenytoin and Kandhasamy et al. (2021) 1) with silk fibroin electrospun fiber are selected to subgroup analysis. The result indicates that, vitamin K treatment was associated with an increased wound healing rate [WMD = 20.11 (95% CI: 16.71, 23.52)]. The heterogeneity of the combined results was very low (I^2^ = 0%, *p* = 0.69). The overall effect test (Z) was 11.58 (*p* < 0.00001). This suggests that vitamin K may be related to promoting wound healing rate, and the concentration of vitamin K and control group’s differences may be the main cause of heterogeneity. The heterogeneity of cell experiments is also significant (I^2^ = 99%, *p* < 0.00001). After removing each study in turn, significant heterogeneity is still observed, which may be related to the types of cells, different control groups, vitamin K concentrations, and forms. The limited number of included studies is also the main source of heterogeneity. The two studies conducted by Rush et al. (2016) selected human telomerase-immortalized corneal epithelial cells. The intervention group used different concentrations of vitamins and receptor tyrosine kinase inhibitors, and the receptor tyrosine kinase inhibitor group was set as the control, the treatment time of vitamin K is 4.5 h. However, the four studies conducted by Pinilla et al. (2014) selected human connective fibroblasts to explore the effect of different concentrations of vitamin K on wound healing, the treatment time of vitamin K is 18 h. The results showed no difference between the 1 mg/L vitamin K3 group and the control group, whereas vitamin K3 significantly reduced the speed of wound healing at 2, 4, and 6 mg/L. Excluding two studies of Rush et al. (2016), the result still showed that vitamin K significantly decelerates the process of wound healing; Excluding Pinilla et al.’s four studies (Pinilla et al., 2014), the result shows that vitamin K significantly improves wound healing rate. This suggests that the concentration of vitamin K may have a great impact on the results. In addition, the difference of cell type and treatment time may also be an important reason for different results. Therefore, the opposite summary results of animal models and cell experiments may be due to the difference in vitamin K concentration. The safe dose and concentration of vitamin K is a problem worthy of our attention, which needs more large-scale and high-quality randomized trials for further research. The reasons lead to different results of animal models and cell experiments may be as follows: in fact, there are some unavoidable factors to add the drug consumption in animal models, such as waste during exercises. So, it is very hard to measure the effective doses. While in cell experiments vitamin K is added into the culture and could interact with cells directly. In cell models, although the researchers use the dose of vitamin K which is calculated based on animal experiments, it may be not suitable for cells. In that, the concentration and bioavailability of drugs will be relatively high, which may be harmful to wound healing; the intervention measures of these animal experimental studies are vitamin K, some of them contain adjuncts, such as silk fibroin. The treatment for animal is the drug laid on the surface of wound, but the vitamin K is used as an addition in cultures in cell experiments; There are also some differences between animal experiments models and cell experiments models. Animal experiments used full-thickness skin wound models. The process of wound healing includes hemostasis, inflammation, proliferation and remodeling, which is a complex process requiring coordination of multiple factors (Wilkinson and Hardman, 2020). Vitamin K plays an important role in wound healing in animal models because of its pharmacological effects such as promoting hemostasis, anti-inflammatory, antioxidant, and promoting cell proliferation and differentiation (Simes et al., 2020). The cell experiments modeling method is to use physical methods to create a cell-free area, mainly to explore the role of vitamin K in promoting cell proliferation and migration, which may also be an important reason for the opposite combined results of animal models and cell experiments. The quality of evidence assessed by GRADE approach are not very satisfactory, because there are few studies on this field at present. However, it provides us a potential method to promote wound healing, and we hope that more relevant studies can be published in the future.

The study of Hemmati et al. (2014) and Amaral et al. (2014) reported the wound tensile strength. The combined results suggested that there was no significant difference in wound tensile strength between vitamin K group and control group. These studies also showed high heterogeneity, which may be related to different wound types and control groups. Of course, the small number of included studies is also a very important reason.

Wounds are common worldwide, and its incidence is also increasing rapidly, which causes a heavy burden on medical care and economy. Based on the survey, about 6.5 million chronic wound patients are reported every year in the United States, and the annual cost of chronic wound treatment is more than 25 billion dollars. Post-traumatic repair is complex, and it is affected by many factors, which is also a great challenge for medical workers. At present, antibiotics are primarily used to treat wounds because of the antibacterial and bactericidal properties, but the abuse of antibiotics will also lead to certain side effects and lose good efficacy. Wound healing therapies can be broadly divided into traditional therapies and modern therapies. Traditional therapies include aloe vera, calendula officinalis, honey, propolis, traditional dressings, silver, phenytoin, etc; Modern therapies include modern dressings, bioengineered skin substitutes, grafts, cell/growth factor therapies etc, (Pereira and Bártolo, 2016; Doshi et al., 2021). In recent years, people have found that vitamin K has certain advantages in wound treatment. Hemmati et al. (2014) studied the application of 1% vitamin K1, 2% vitamin K1 and 1% phenytoin in the treatment of wound healing. The results showed that 1% vitamin K1 and 2% vitamin K1 were more effective than 1% phenytoin. In addition, the results of this meta-analysis of animal models also suggest that vitamin K has a certain effect on wound healing. The combination of vitamin K with these traditional and modern therapies may have a greater effect on wound healing, which also gives us an idea to develop a new drug in the future. In addition, the different bioavailability, therapeutic dose and duration of drugs may also have a great impact on the therapeutic effect (Dludla et al., 2022). Relevant studies have shown that MK7, a subtype of vitamin K2, is the most effective in terms of bioavailability and biological effects (Cirilli et al., 2020; Cirilli et al., 2022). Trans MK7 shows a higher ability to carboxylate Gla protein than cis MK7 (Cirilli et al., 2022). This suggests that the type and chemical structure of vitamins are also very important factors. Therefore, more in-depth research is needed to decode the effect of vitamins on wound healing.

**FIGURE 5:**
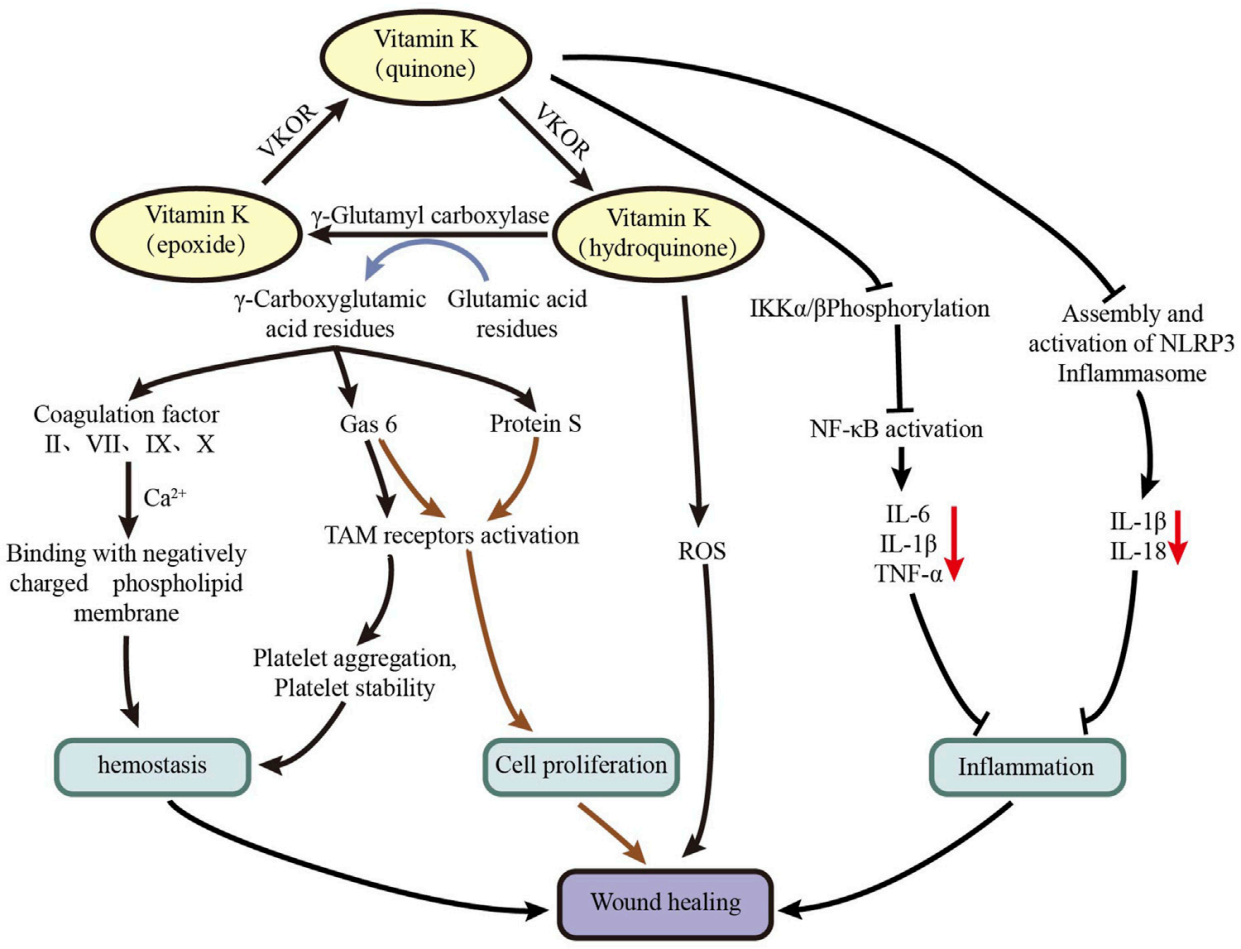
Possible mechanism of vitamin K for wound healing.

The mechanism of vitamin K in the treatment of wound healing can be attributed to a variety of reasons can be attributed to a variety of reasons (Figure 5): vitamin K often exists in the form of vitamin K quinone, which is converted into vitamin K hydroquinone under the action of vitamin K oxidoreductase. Under the action of γ-glutamic acid carboxylase, vitamin K hydroquinone is converted into vitamin K epoxide, and at the same time, inactive glutamic acid residues of coagulation factors (II, VII, IX, X), protein S, and Gas6 is converted into active γ-carboxyglutamic acid. Under the action of vitamin K oxidoreductase, vitamin K epoxide is converted into vitamin K quinone, thereby forming the vitamin K cycle (Schurgers and Spronk, 2014). 1) Activated coagulation factors II, VII, IX, and X provide effective chelating sites for calcium ions, and they can easily combine with negatively charged phospholipid membrane to promote hemostasis (Kaesler et al., 2021). In addition, Gas6 can activate Tyro3, Axl, and Mer (TAM) receptors, which is conducive to platelet aggregation and stability, thereby promoting hemostasis (van der Meer et al., 2014). 2) Protein S and Gas6 can activate TAM receptors to promote cell proliferation, thereby accelerating wound healing (Hafizi and Dahlbäck, 2006; van der Meer et al., 2014). 3) Vitamin K hydroquinone can be used as an effective antioxidant to remove excess active oxygen from damaged parts (Vervoort et al., 1997). 4) Vitamin K can inhibit inhibitor of kappa-B kinase (IKK) α/β phosphorylation to inhibit nuclear factor kappa-B (NF-κB) activation, thereby reducing the expression of IL-6, IL-1β, TNF-α, and other inflammatory factors, which play a role in inhibiting inflammatory response (Li et al., 2018). Vitamin K can also inhibit the expression of IL-1β and IL-18 by inhibiting the assembly and activation of NLRP3 inflammasome, which plays a role in inhibiting inflammatory response (Huang et al., 2020; Zheng et al., 2021). The research of Osman et al. (Osman and Amin, 2020) showed that the combination of vitamin K and Mebootment may promote wound healing by promoting the expression of transforming growth factor β (TGF-β) and platelet-derived growth factor (PDGF). The results of the corneal epithelial injury mouse model test of Rush et al. (2016) show that vitamin K3 can promote corneal epithelial wound healing *in vivo*. A randomized controlled clinical trial of Pazyar et al. (2019) showed that the wound area of the vitamin K group was significantly reduced on the 4th day after injury compared with the control group, but no significant difference was observed on the 7th day. Furthermore, no significant difference in healing time was found between the two groups.

In this study, we also found that some studies reported other indicators that were not included in the meta-analysis, such as epithelialization time, hydroproline content, growth factors and cell proliferation efficiency, to indicate the effect of vitamin K on wound healing (Table 4). These studies used rats and cells as research objects to test the effect of vitamin K. The research of Ambrożewicz et al. (2019) showed that vitamin K could reduce the adverse effects of hydroxyapatite-based biomaterials and promote the repair of bone damage because of its evident bone-inducing, antioxidant, and anti-inflammatory properties. Another study showed that the increase in wound severity was associated with a decrease in the intake of nutrients such as vitamin K (Wojcik et al., 2011).

**TABLE 4:** Characteristics of Vitamin K for wound healing.

Studies	Characteristics	Results		Materials	Sampling time
		Intervention control			
Hemmati et al. (2014)	GE (days)	14.7 ± 0.8	21.7 ± 0.5	albino rats (M)	--
	GE (days)	15 ± 0.7	21.7 ± 0.5		--
	Hydroxyproline (μg/g)	1100 ± 40	878 ± 18		after complete healing
	Hydroxyproline (μg/g)	1074 ± 34	878 ± 18		after complete healing
Rush et al. (2016)	Cell Growth (fold over basal)	2.03 ± 0.32	1.71 ± 0.24	HRE cells	20 h
Kandhasamy et al. (2021)	Quantification of cells	581.6 ± 13.93	431.83 ± 13.93	NIH 3T3 cells	6 days
	Cell Migration (number per field)	695.06 ± 22.73	375.86 ± 13.64		24 h
	Quantification of cells	425.64 ± 12.82	179.49 ± 12.82	HFF1 cells	6 days
Osman and Amin. (2020)	TGF-β (pg/ml)	56.5 ± 1.29	35.75 ± 4.856	albino rats (M)	14 days
	TGF-β (pg/ml)	40.5 ± 4.795	35.5 ± 1.29		14 days
	TGF-β (pg/ml)	43 ± 2.16	3.5 ± 0.577		14 days
	PDGF (pg/ml)	0.386 ± 0.354	0.097 ± 0.02		21 days

GE, growth of epidermis; HRE, human retinal endothelial; NIH3T3, mouse embryonic fibroblast; HFF1, human skin fibroblast.

This study has also some limitations. First, the number of studies included in this meta-analysis and the number of literatures included in each group are limited. In addition, the heterogeneity is significant, which may have some effects on our results. Second, given the small number of literatures included in each group, we did not conduct a publication bias assessment. Moreover, the form and composition of vitamin K in these study intervention groups are quite different, such as the use of different concentrations of vitamin K and the introduction of biomaterials. Different control groups and treatment time may also affect the results. The current research in this field is preliminary, and it is difficult to draw any conclusion about the use of vitamin K in wound healing. It is hoped that more domestic and foreign scholars can invest more samples in research to explore the effect of vitamin K on wound healing and its safe dose and concentration. With the publication of more studies in this area, more definite results can be provided.

## Conclusion

In this meta-analysis, we found that vitamin K may increase wound healing rate in animal models, but opposite results were obtained in cell tests. Therefore, vitamin K has a certain effect on wound healing, but its role remains unknown, which needs to be studied on a larger scale. In addition, the concentration, safe dose, treatment time, type and chemical structure of vitamin K deserve further study.

## Supplementary material

Supplementary Material S1 Detailed search strategy.

Supplementary Material S2 PRISMA 2020 Checklist.

Supplementary Material S3 AMSTAR 2 Checklist.

Supplementary Material S4 Original data of this meta-analysis.

Supplementary Material S5 Not included articles
