# Supplementary material for: Effect of vitamin K on wound healing: A systematic review and meta-analysis based on preclinical studies
Source: Front Pharmacol. 2022 Dec 2;13:1063349. doi: 10.3389/fphar.2022.1063349 (PMC9755209; doi:10.3389/fphar.2022.1063349)
Supplement: Supplementary file 1 [file DataSheet4.DOCX]

**S-Table1. Data of wound contraction percentages extracted from included studies in rat model.**

| Study(rat) | intervention | | total | control | | total |
| --- | --- | --- | --- | --- | --- | --- |
|  | Mean | SD |  | mean | SD |  |
| *Hemmati et al 2014(1)  *Hemmati et al 2014(2)  *Hemmati et al 2014(3)  *Hemmati et al 2014(4)  Kandhasamy et al 2021(1)  Kandhasamy et al 2021(2) | 50.68  40.92  50.68  40.92  81.3  81.3 | 4.7  4.34  4.7  4.34  4.21  4.21 | 8  8  8  8  6  6 | 18.87  18.87  31.52  31.52  60.68  19.9 | 3.25  3.25  6.88  6.88  3.16  3.66 | 8  8  8  8  6  6 |

**S-Table2. Data of wound contraction percentages extracted from included studies in cell model.**

| Study(cell) | intervention | | total | control | | total |
| --- | --- | --- | --- | --- | --- | --- |
|  | Mean | SD |  | mean | SD |  |
| **Δ**Pinilla et al 2014(1)  **Δ**Pinilla et al 2014(2)  **Δ**Pinilla et al 2014(3)  **Δ**Pinilla et al 2014(4)  Rush et al 2016(2)  Rush et al 2016(3) | 28.82  -0.45  -39.17  -32.99  83.73  78.64 | 9.84  14.2  17.33  6.7  1.38  2.27 | 12  12  12  12  4  4 | 44.57  44.57  44.57  44.57  73.79  73.79 | 18.03  18.03  18.03  18.03  2.59  2.59 | 12  12  12  12  4  4 |

| Study(rat) | intervention | | total | control | | total |
| --- | --- | --- | --- | --- | --- | --- |
|  | mean | SD |  | mean | SD |  |
| Hemmati et al 2014(1) | 1200 | 40 | 8 | 987 | 33 | 8 |
| Hemmati et al 2014(2) | 1170 | 34 | 8 | 987 | 33 | 8 |
| Hemmati et al 2014(3) | 1200 | 40 | 8 | 1229 | 60 | 8 |
| Hemmati et al 2014(4) | 1170 | 34 | 8 | 1229 | 60 | 8 |
| *Amaral et al 2014 | 228.5 | 37.4 | 4 | 208.3 | 15.2 | 4 |

**S-Table3. Data of wound tensile strength extracted from included studies in rat model.**

***The data has been converted by some simple arithmetic. The original has been presented below.**

| study | groups | wound area/ initial area | Sample number |
| --- | --- | --- | --- |
| Hemmati et al 2014(1) | Intervention | 49.32±4.7 | 8 |
| Hemmati et al 2014(2)  Hemmati et al 2014(3)  Hemmati et al 2014(4) | Control  Intervention  Control  Intervention  Control  Intervention  Control | 81.13±3.25  59.08±4.34  81.13±3.25  49.32±4.7  68.48±6.88  59.08±4.34  68.48±6.88 | 8  8  8  8  8  8  8 |

**ΔThe data has been converted by some simple arithmetic. The original has been presented below.**

| study | groups | wound area(mm^2^) | initial area (mm^2^) | Sample number |
| --- | --- | --- | --- | --- |
| Pinilla et al 2014(1)  Pinilla et al 2014(2)  Pinilla et al 2014(3)  Pinilla et al 2014(4) | Intervention  Control  Intervention  Control  Intervention  Control  Intervention  Control | 0.405±0.056  0.332±0.108  0.672±0.095  0.332±0.108  0.771±0.096  0.332±0.108  0.774±0.039  0.332±0.108 | 0.569±0.099  0.599±0.118  0.669±0.120  0.599±0.118  0.554±0.162  0.599±0.118  0.582±0.110  0.599±0.118 | 12  12  12  12  12  12  12  12 |

wound contraction percentages= [(initial area- wound area)/ initial area]×100%.

***The data has been converted by some simple arithmetic. The original has been presented below.**

| study | groups | bursting pressure(mmHg) | Sample number |
| --- | --- | --- | --- |
| Amaral et al 2014 | Intervention | 171.392±28.029 | 4 |
|  | Control | 156.266±11.387 | 4 |
