# Supplementary material for: Effect of vitamin K on wound healing: A systematic review and meta-analysis based on preclinical studies
Source: Front Pharmacol. 2022 Dec 2;13:1063349. doi: 10.3389/fphar.2022.1063349 (PMC9755209; doi:10.3389/fphar.2022.1063349)
Supplement: Supplementary file 3 [file DataSheet5.DOCX]

| **Studies** | **Title** | **Country** | **Reasons for excluded** |
| --- | --- | --- | --- |
| Varsha MK et al.  (2015)[1] | Vitamin K1 alleviates streptozotocin-induced type 1 diabetes by mitigating free radical stress, as well as inhibiting NF-κB activation and iNOS expression in rat pancreas | India | uncorrelated to effect of vitamin K on wound healing |
| Weng Sheji et al.  (2019)[2] | Effects of combined menaquinone-4 and PTH(1-34) treatment on osetogenesis and angiogenesis in calvarial defect in osteopenic rats | China | uncorrelated to effect of vitamin K on wound healing |
| Esmaeili A et al.  (2013)[3] | Characterization of nanocapsules containing Elaeagnus angustifolia L. extract prepared using an emulsion-diffusion process | Iran | uncorrelated to effect of vitamin K on wound healing |
| Kovács IB et al. (1967)[4] | Effect of vitamin K on connective tissue metabolism | Netherlands | uncorrelated to effect of vitamin K on wound healing |
| Wang Wei et al.  (2002)[5]  Cong Hongxia et al. (2020)[6]  Osman, Shokhan  et al. (2020)[7] | Clinical observation on 100 cases of postoperative pain of anal-intestinal diseases  treated with vitamine K blocking at Changqiang point  Repairing Effects of Duck Oil Diglyceride Combined with Vitamin K1 on Dextran Sodium Sulfate-Induced Ulcerative Colitis Injury in Mice  The effect of vitamin K on the wound healing process in rat skin achieved by common wound dressing agents | China  China  Iraq | not report wound healing rate/days or tensile strength  not report wound healing rate/days or tensile strength  not report wound healing rate/days or tensile strength |

**The 7 excluded articles**

[1] Varsha MK, Thiagarajan R, Manikandan R, et al.Vitamin K1 alleviates streptozotocin-induced type 1 diabetes by mitigating free radical stress, as well as inhibiting NF-κB activation and iNOS expression in rat pancreas.Nutrition,2015,31(1):214-222

[2] Weng SJ, Xie ZJ, Wu ZY, et al.Effects of combined menaquinone-4 and PTH(1-34) treatment on osetogenesis and angiogenesis in calvarial defect in osteopenic rats.Endocrine,2019,63(2):376-384

[3] Esmaeili A, Niknam S.Characterization of nanocapsules containing Elaeagnus angustifolia L. extract prepared using an emulsion-diffusion process.Flavour and Fragrance Journal,2013,28(5):309-315

[4] Kovács IB, Görög P, Szporny L, et al.Effect of vitamin K on connective tissue metabolism.Biochemical pharmacology,1967,16(3):575-578

[5] Wang W, Zhang J.Clinical observation on 100 cases of postoperative pain of anal-intestinal diseases treated with vitamine K blocking at Changqiang point.Journal of Traditional Chinese Medicine,2002,22(4):280-281

[6] Cong H, Wang B, Ge W, et al.Repairing Effects of Duck Oil Diglyceride Combined with Vitamin K1 on Dextran Sodium Sulfate-Induced Ulcerative Colitis Injury in Mice.Chinese Journal of Animal Nutrition,2020,32(9):4376-4385

[7] Osman S, Amin Z.The effect of vitamin K on the wound healing process in rat skin achieved by common wound dressing agents.Zanco Journal of Medical Sciences,2020,24(1):107-116
