# Supplementary material for: Effect of vitamin K on wound healing: A systematic review and meta-analysis based on preclinical studies
Source: Front Pharmacol. 2022 Dec 2;13:1063349. doi: 10.3389/fphar.2022.1063349 (PMC9755209; doi:10.3389/fphar.2022.1063349)

**PubMed:（113）**

(((((((((("Wound Healing"[Mesh]) OR (wound healing[Title/Abstract])) OR (wound healings[Title/Abstract])) OR (healing, wound[Title/Abstract])) OR (healings, wound[Title/Abstract])) OR (regeneration[Title/Abstract])) OR (injury repair[Title/Abstract])) OR (wound repair[Title/Abstract])) OR (re epithelialization[Title/Abstract])) OR (wound epithelialization[Title/Abstract])) AND (((((((((((((((("Vitamin K"[Mesh]) OR (Vitamin K[Title/Abstract])) OR (VK[Title/Abstract])) OR (Vitamin K1[Title/Abstract])) OR (Vitamin K2[Title/Abstract])) OR (Vitamin K3[Title/Abstract])) OR (Vitamin K 1[Title/Abstract])) OR (Phylloquinone[Title/Abstract])) OR (Phytonadione[Title/Abstract])) OR (Phytomenadione[Title/Abstract])) OR (Konakion[Title/Abstract])) OR (Vitamin K 2[Title/Abstract])) OR (Menaquinone[Title/Abstract])) OR (menatetrenone[Title/Abstract])) OR (Vitamin K 3[Title/Abstract])) OR (Menadione[Title/Abstract]))


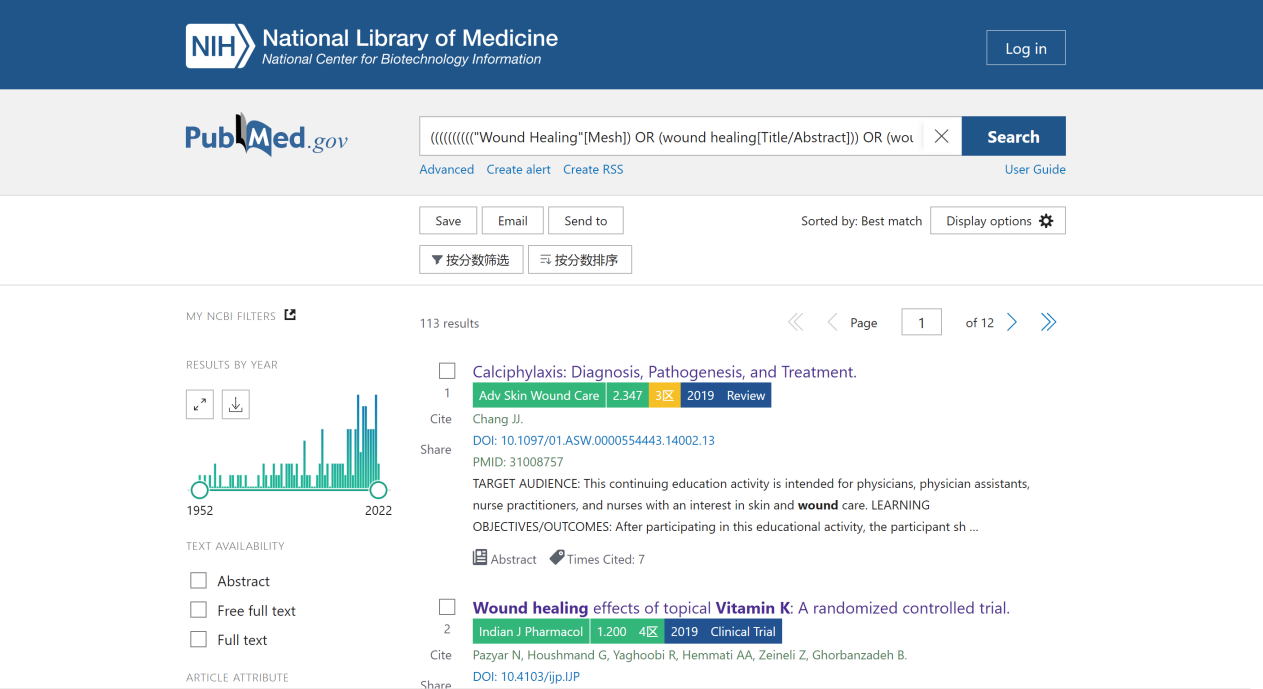


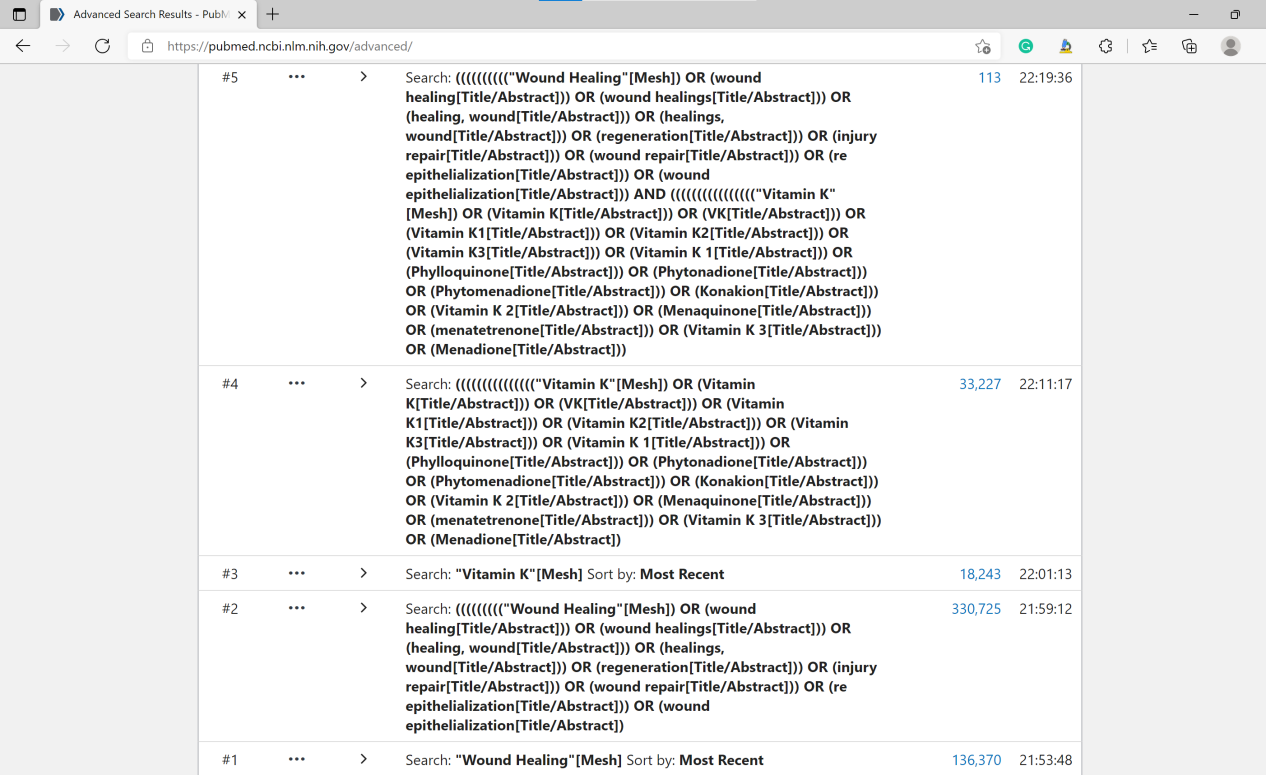


**EMBASE:（227）**


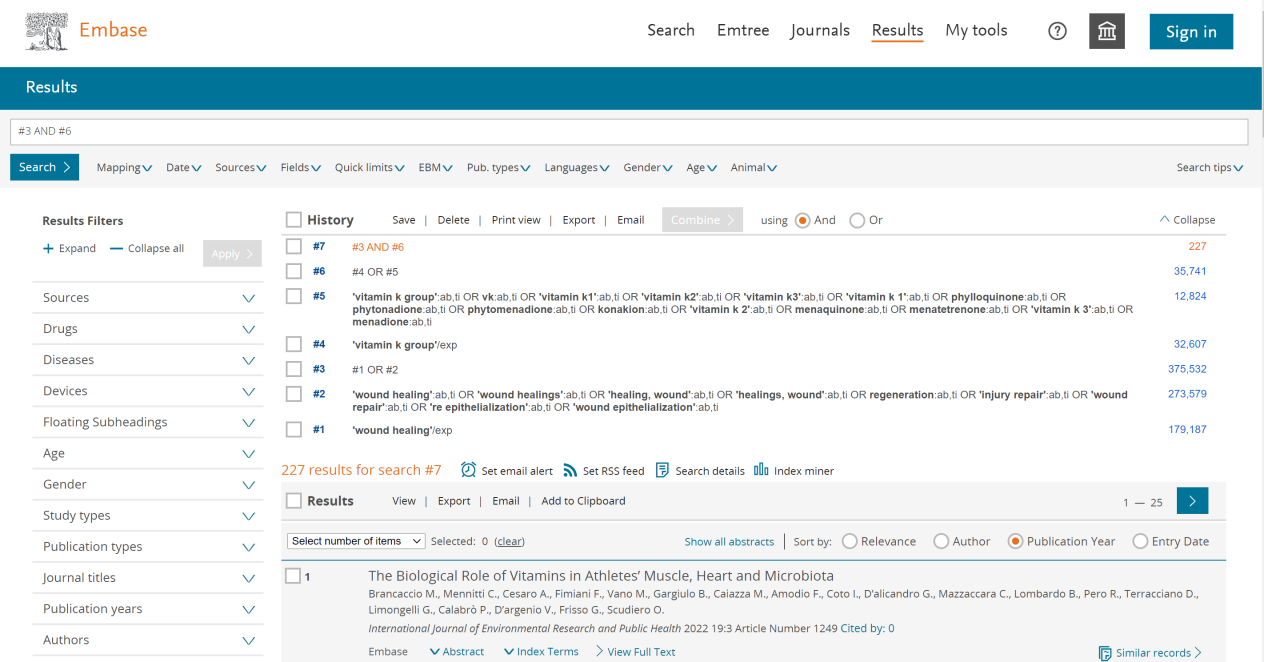


**Web of Science:(718)**


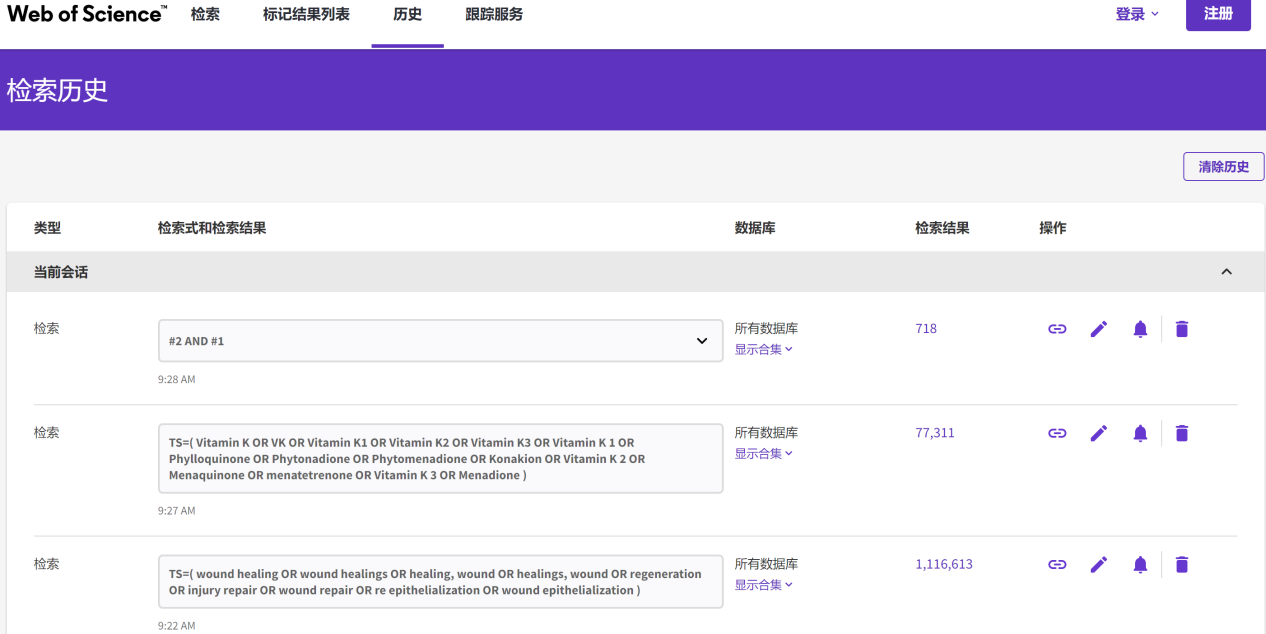


**the Cochrane Library:(15)**


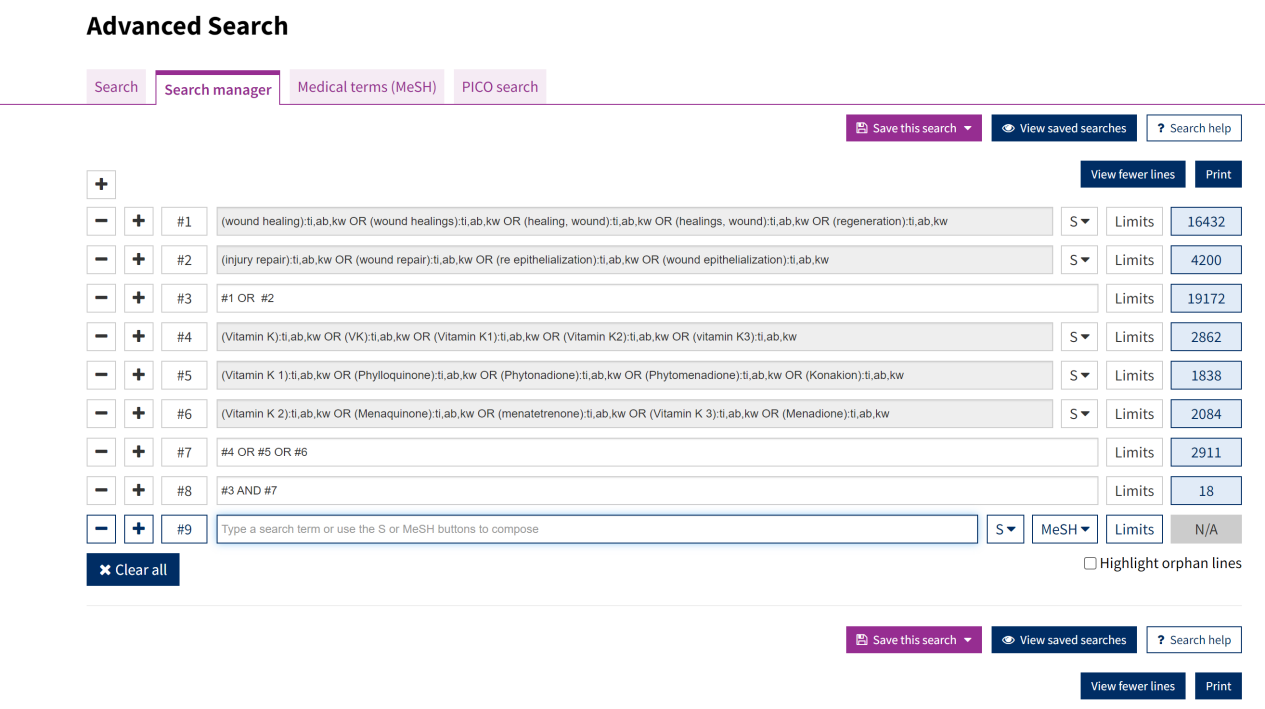


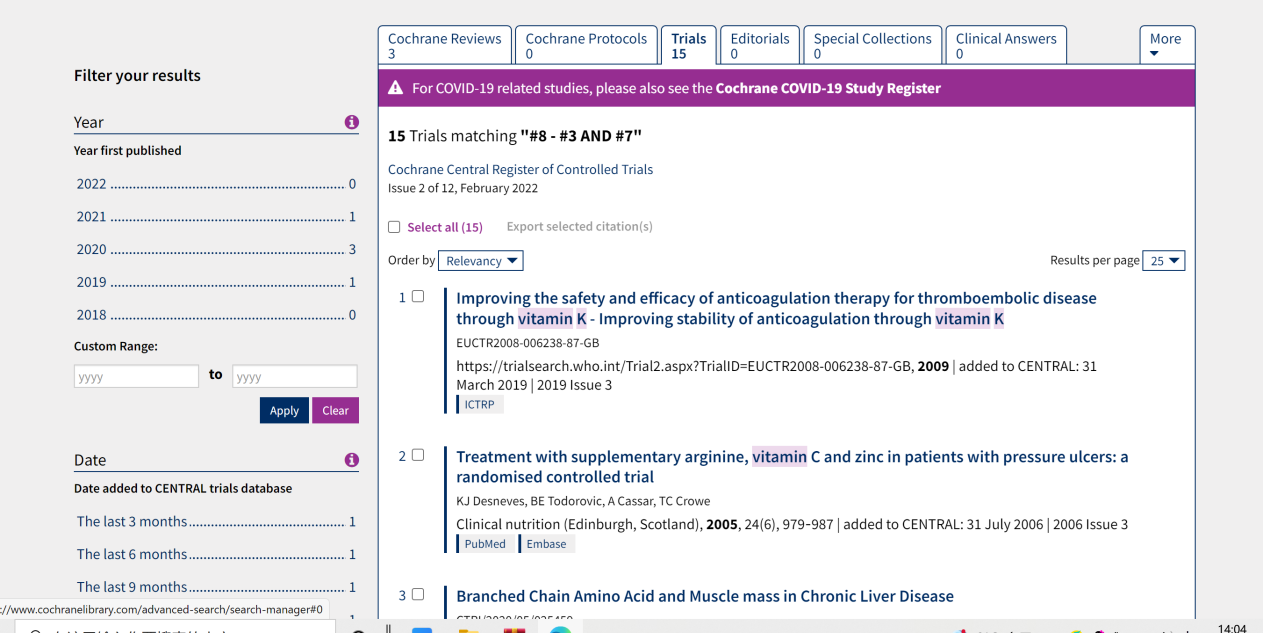


**CNKI:（7）**


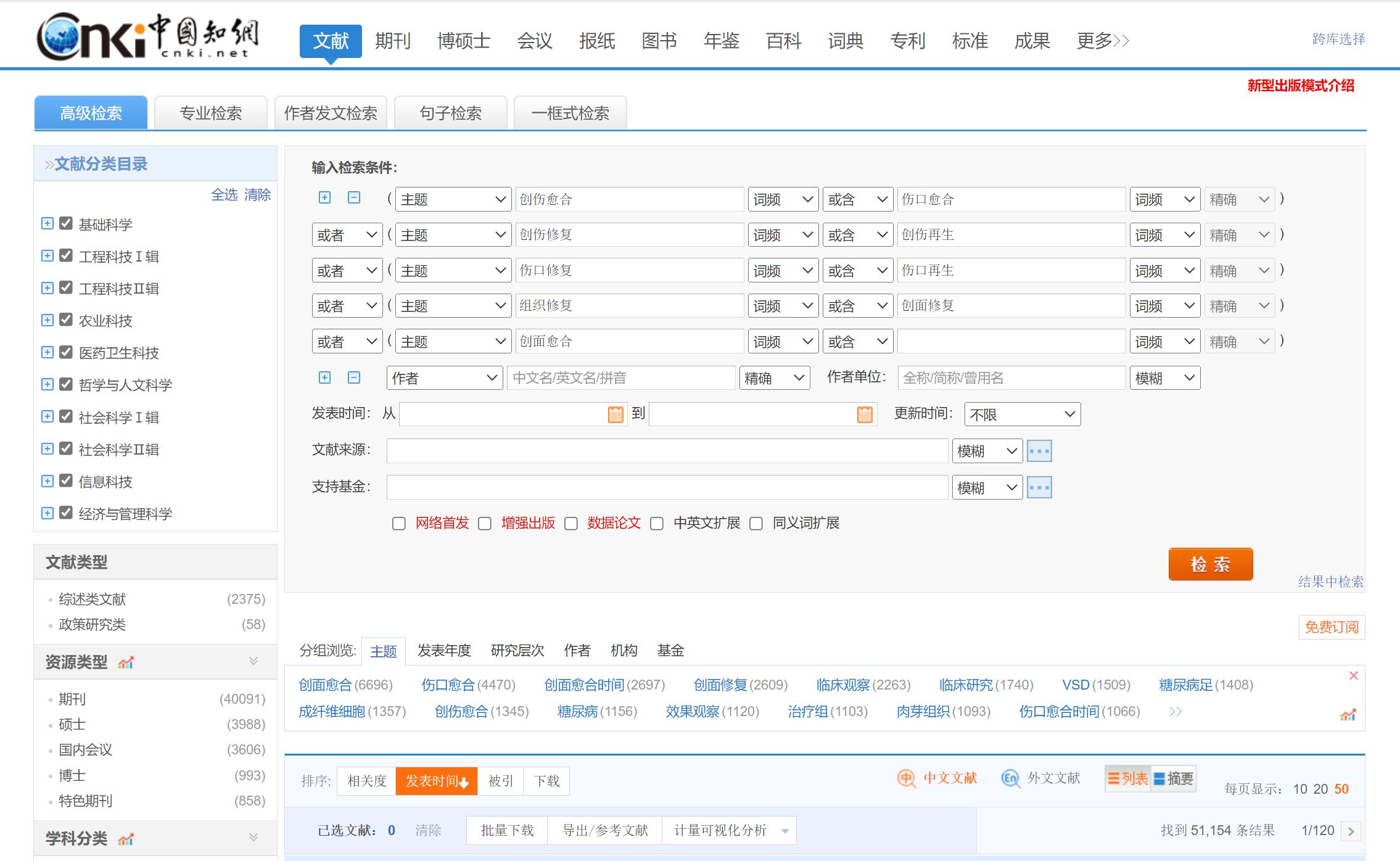


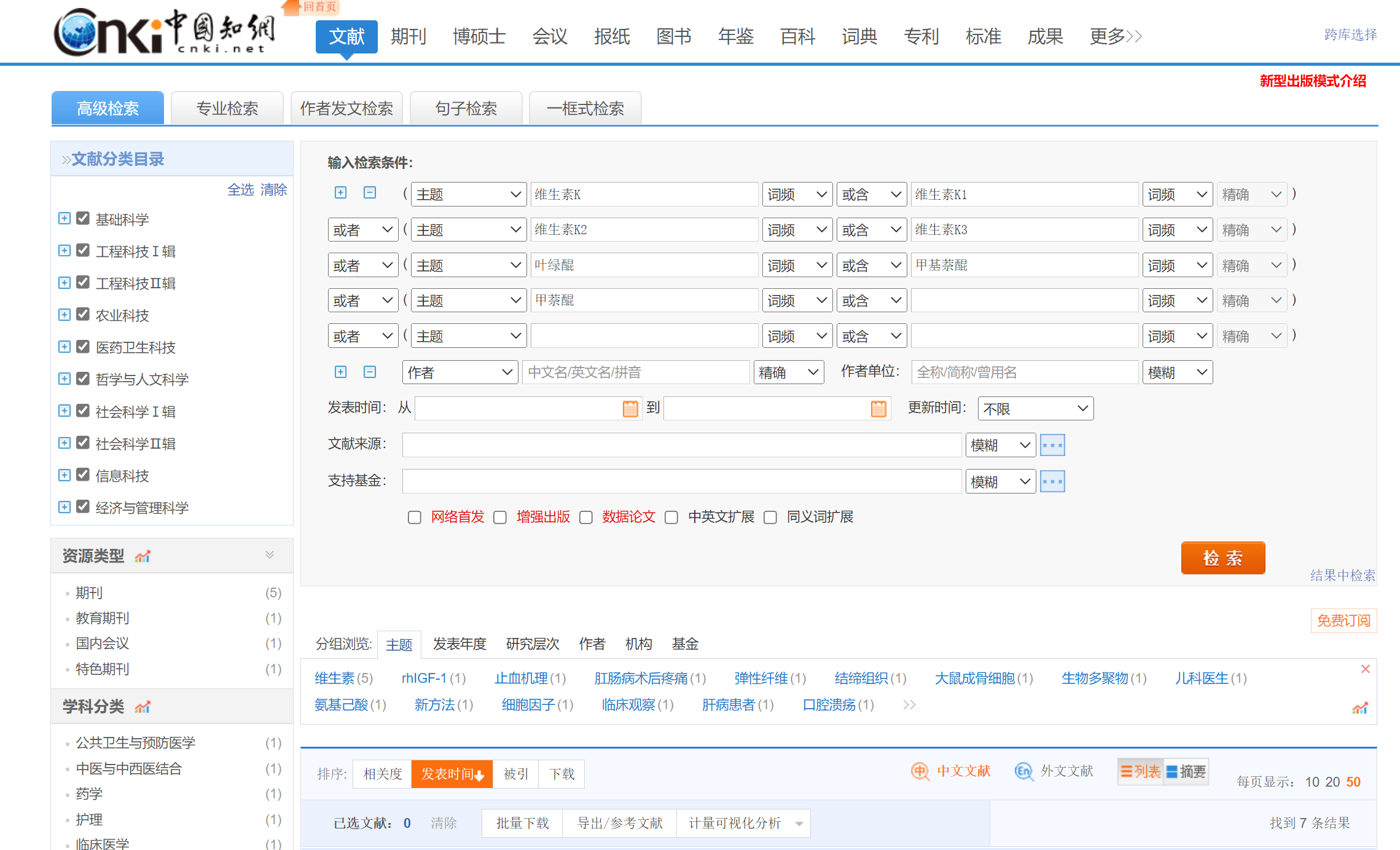


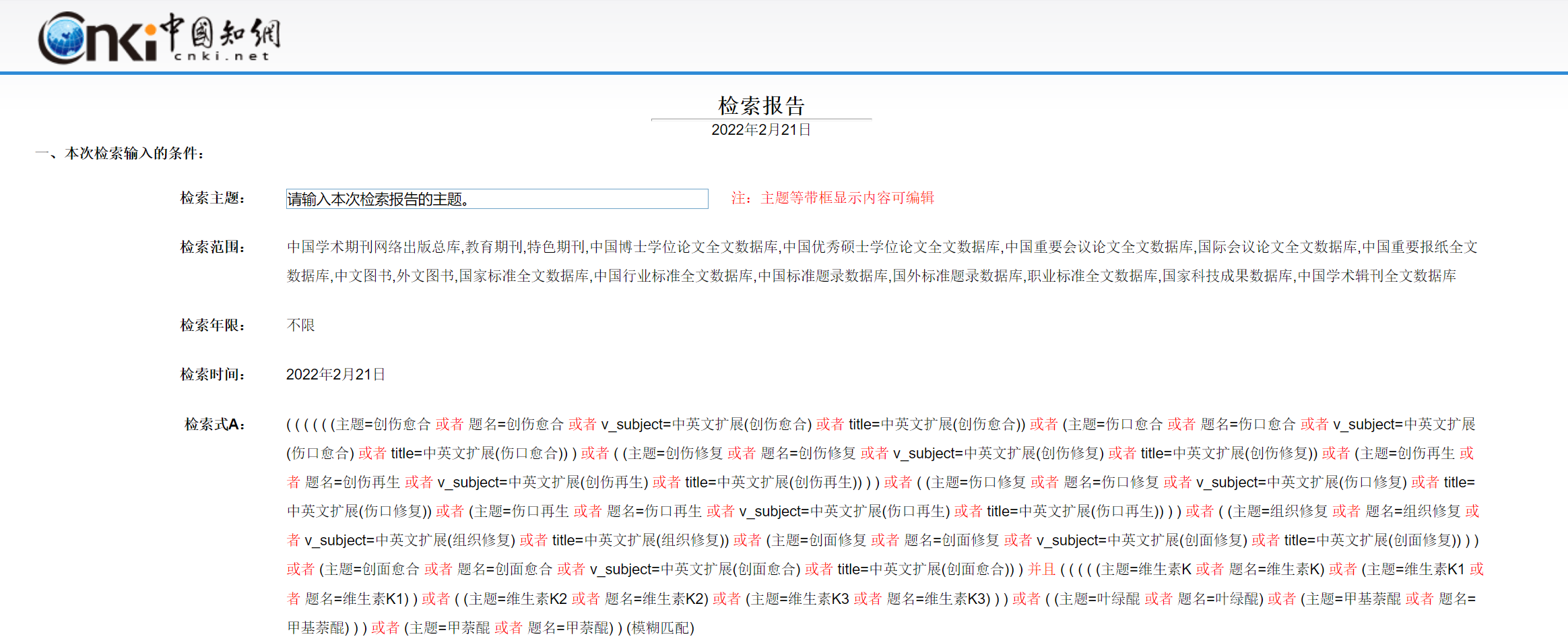


**VIP: （1）**


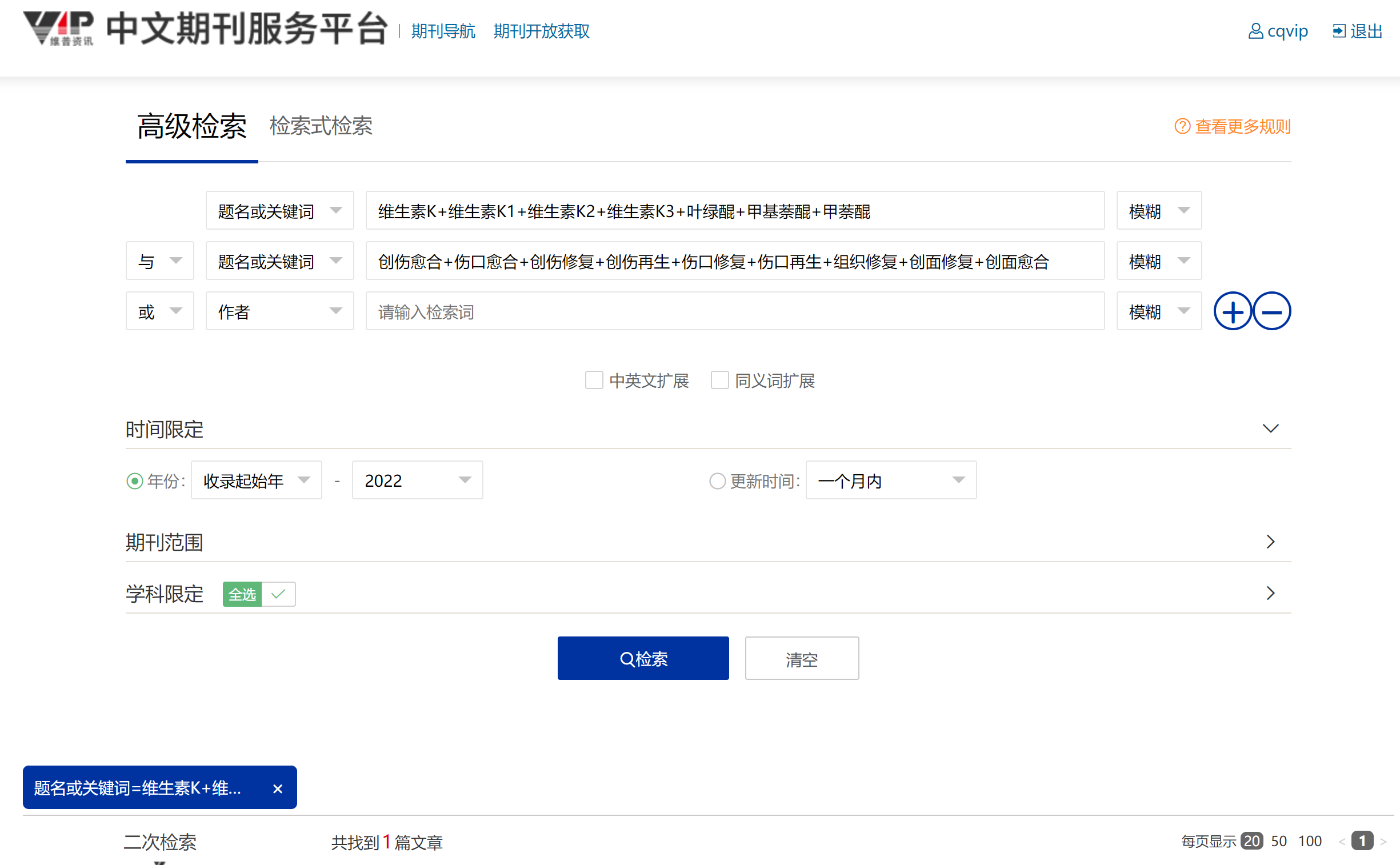


**WanFang:(0)**


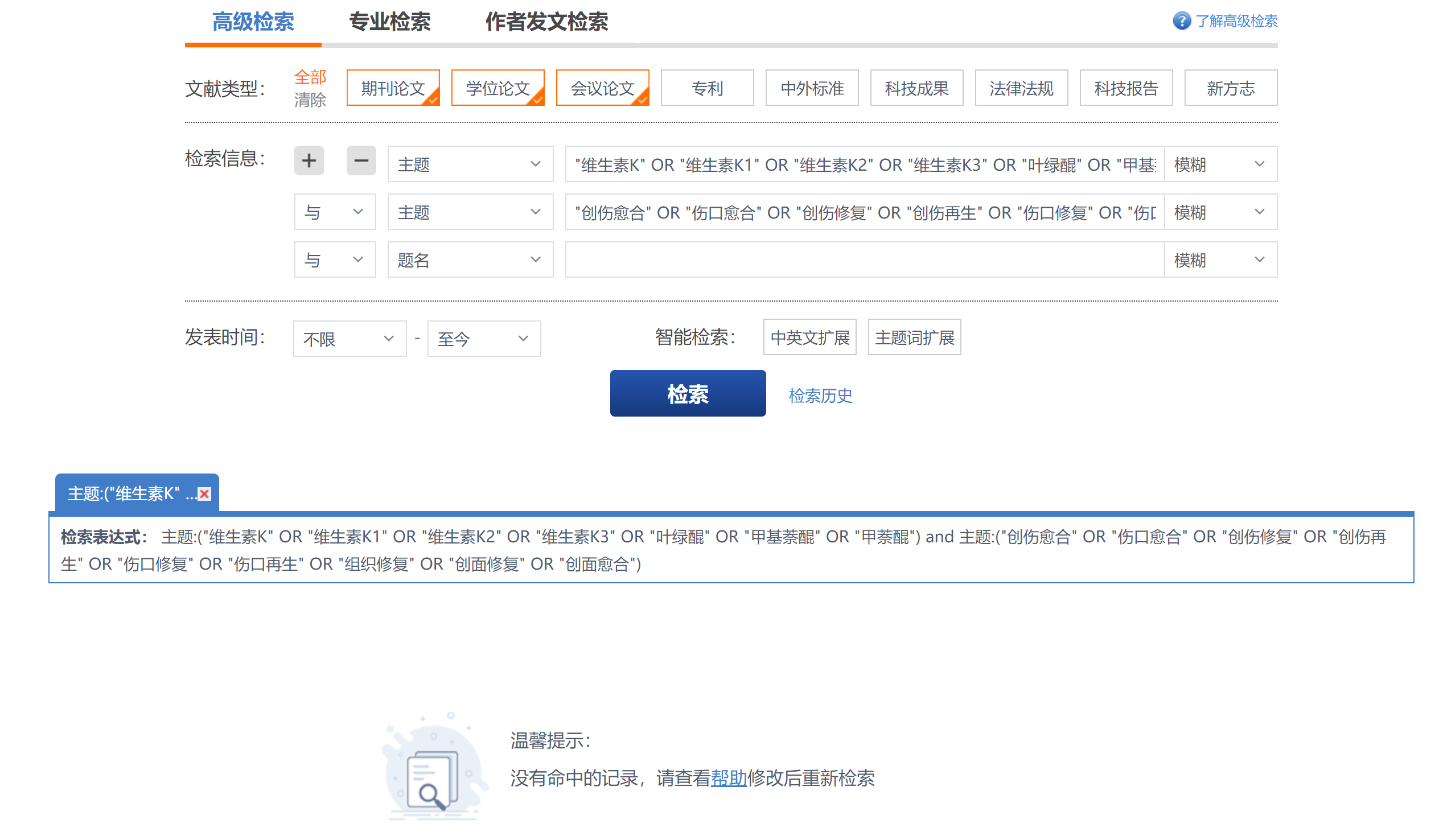

Supplement: Supplementary file 5 [file DataSheet2.DOCX]
